# Results Reporting and Early Termination of Childhood Obesity Trials Registered on ClinicalTrials.gov

**DOI:** 10.3389/fped.2022.860610

**Published:** 2022-03-24

**Authors:** Xinyi Wang, Youlin Long, Liu Yang, Jin Huang, Liang Du

**Affiliations:** ^1^Chinese Evidence-Based Medicine Center, West China Hospital, Sichuan University, Chengdu, China; ^2^Medical Device Regulatory Research and Evaluation Centre, West China Hospital, Sichuan University, Chengdu, China; ^3^West China Medical Publishers, West China Hospital, Sichuan University, Chengdu, China

**Keywords:** childhood obesity, early termination, results reporting, underreporting, ClinicalTrials.gov

## Abstract

**Objective:**

Childhood obesity is one of the most severe challenges of public health in the twenty-first century and may increase the risk of various physical and psychological diseases in adulthood. The prevalence and predictors of unreported results and premature termination in pediatric obesity research are not clear. We aimed to characterize childhood obesity trials registered on ClinicalTrials.gov and identify features associated with early termination and lack of results reporting.

**Methods:**

Records were downloaded and screened for all childhood obesity trials from the inception of ClinicalTrials.gov to July 29, 2021. We performed descriptive analyses of characteristics, Cox regression for early termination, and logistic regression for lack of results reporting.

**Results:**

We identified 1,312 trials registered at ClinicalTrials.gov. Among clinicalTrials.gov registered childhood obesity-related intervention trials, trial unreported results were 88.5 and 4.3% of trials were prematurely terminated. Additionally, the factors that reduced the risk of unreported outcomes were US-registered clinical studies and drug intervention trials. Factors associated with a reduced risk of early termination are National Institutes of Health (NIH) or other federal agency funding and large trials.

**Conclusion:**

The problem of unreported results in clinical trials of childhood obesity is serious. Therefore, timely bulletin of the results and reasons for termination remain urgent aims for childhood obesity trials.

## Introduction

Childhood obesity is one of the most severe challenges of public health in the twenty-first century, affecting ~41 million children under the age of 5 years (1, 2). The prevalence of overweight and obesity in children started to rise at the end of the 1980's and continues to rise in some countries (3). Childhood obesity increases the risk of having obesity in adulthood and health problems such as diabetes and cardiovascular diseases (4–10). Furthermore, growing prevalence of obesity can place a substantial cost burden to the health-care system. The overall economic cost of obesity worldwide was estimated at $2.0 trillion US dollars in 2012, with an estimated 5.1 billion a year spent on obesity-related diseases by the National Health Service (NHS) (11–13).

Clinical trials rely on the participation of volunteers, involving massive investment of human, material and financial resources (14). Meanwhile, trial researchers have an obligation to participants not only to minimize potential harm, but also to conduct trials scrupulously, and to publicly report their findings in time. Unpublished trial results could violate ethical obligations to participants and compromise existing medical evidence (15). Policies have been enacted to improve the transparency and accountability of trials in recent years. The International Committee of Medical Journal Editors has required registration of interventional trials in a public trials registry [such as ClinicalTrials.gov, a registry of clinical trials implemented in 2000 by the National Library of Medicine-National Institutes of Health (NIH)] as a condition for publication since July 1, 2005 (16). Furthermore, the Food and Drug Administration Amendments Act of 2007 (FDAAA) required that summary results of certain trials should be submitted within 1 year of final collection of data for the primary outcome, including whether the trials concluded according to the predetermined protocol or were discontinued (17). However, despite these increased ethical and legislative requirements, the discontinuation and unreported results of clinical trials remain common.

Although a limited number of studies have cross-sectional descriptions of pediatric trials (18–20), they have not further identified the factors of unreported results and premature termination in pediatric obesity research. In addition, analyses of trial discontinuation and result reporting have primarily focused on adult populations, and the prevalence of these outcomes for pediatric randomized controlled trials (RCTs) remains less well defined (21). We suppose that pediatric clinical trials are easier to stop early because of ethical and recruitment issues. Therefore, we aimed to investigate the factors of results reporting and premature termination of current childhood obesity trials registered in ClinicalTrials.gov and make corresponding recommendations.

## Materials and Methods

### Data Source

We used the terms “obesity,” “overweight,” “excess weight,” and “body mass index,” searched on ClinicalTrials.gov. To identify pediatric clinical studies, we used the “Age Group: birth-17” criteria. In the retrieval studies, the Age Group field included three types: (Child), (Child, Adult) and (Child, Adult, Older Adult) (18). Records were downloaded on July 29, 2021, for all childhood obesity trials submitted to ClinicalTrials.gov.

### Study Selection

The childhood obesity clinical trial dataset was restricted to interventional studies registered up to July 29^th^. Interventional studies are defined by ClinicalTrials.gov as those in which an investigator assigns an intervention (including diagnostic, therapeutic or other types of interventions) based on a protocol. Noninterventional or observational trials were excluded because they are not currently required to be registered at ClinicalTrials.gov. Each trial was independently screened by two investigators, and disagreements were resolved by consensus or third-party adjudication.

### Data Collection

The following data from each eligible trial were independently extracted by two investigators: general and design characteristics, country, interventions, study status, the results reporting, funding source and date. The duration of the trial was calculated from the beginning of the investigation to primary completion (22). Early discontinuation was defined as a study status including suspended, terminated, or withdrawn (23). In analyzing the reporting of results, we used the “primary completion date” which was interpreted as the date on which the primary outcome data for the last enrolled patient was collected (24). We put off the primary completion date by 1 year, as both NIH regulations and trial reporting policies required sponsors or researchers to submit results data within 1 year of the primary completion date (22, 25). Considering that some of these trials might still publish their results if their results could be reported within 1 year, we would only analyze results reporting for trials completed before July 29, 2020.

### Statistical Analysis

Descriptive statistics were used to summarize trial data with frequencies and percentages. Furthermore, categorical variables were presented as frequencies and percentages. Univariable and multivariable Cox and logistic regression analyses were performed to investigate factors associated with early discontinuation and reporting results, respectively. In regression analysis, “Enrollment: 0-100”, “country: United States”, “Interventions: Drug”, “Funded: Non-industry”, “Allocation: Non-randomized”, “Masking: Yes”, “Primary Purpose: Treatment” was used as the reference group respectively. The hazard ratio (HR)/odds ratio (OR) and 95% confidence interval (CI) were reported. Then, subgroup analysis was conducted, and the trials were divided into two groups according to “both adults and children” and “children only,” and Cox and Logistic regression were performed respectively. For the statistical analysis, the significance level was set at 0.05, and the analysis was performed using STATA 16 software.

## Results

### Characteristics for All Studies

There were 384,836 clinical trials registered in ClinicalTrials.gov as of July 29, 2021.

**Figure 1:**
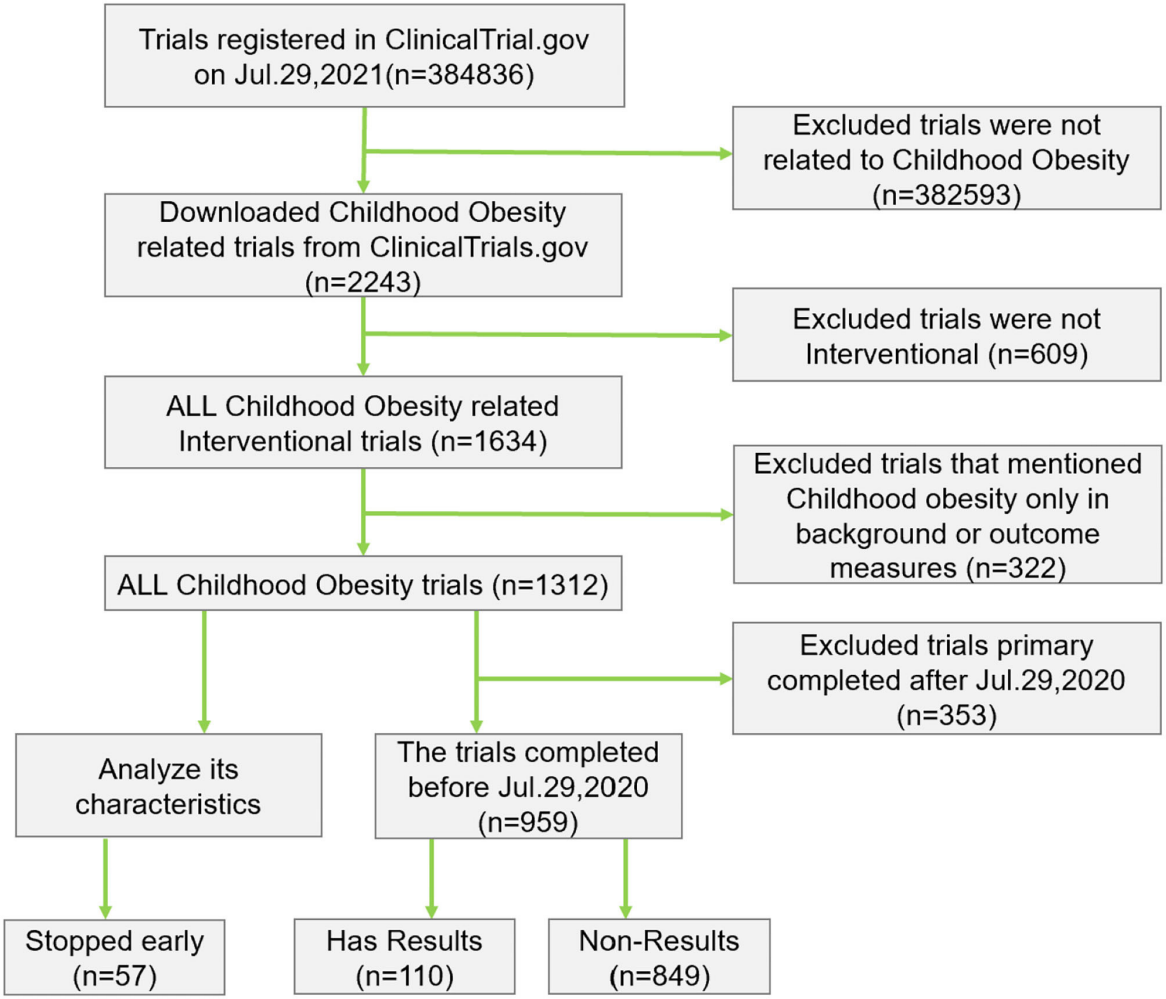
Flowchart of childhood obesity trials.

A total of 2,243 clinical trials were downloaded on childhood obesity among these trials. We excluded 609 trials because they were not interventional. Of the remaining 1,634 interventional trials within this period, we identified 1,312 trials after manual review (Figure 1).

Most trials included both males and females (*n* = 1163, 88.6%) (Table 1). 58.6% included only pediatric patients. The United States ranks first among 58 countries in the number of registered childhood obesity trials (51.7%). A total of 24.1% of the trials were completed within 1 year. Small studies involving <100 patients comprised 50.1% of childhood obesity trials. Only 16.9 and 6.2% of the childhood obesity trials were funded by the NIH and industries, respectively.

**Table 1:** General characteristic of childhood obesity trials (*n* = 1,312).

**Items**	**Detail**	**Number**	**Percent**
Gender	All	1,163	88.6
	Female	126	9.6
	Male	21	1.6
Age	Child	769	58.6
	Child, Adult	329	25.1
	Child, Adult, Older Adult	214	16.3
Start date	Before 2000	14	1.1
	2001–2010	382	29.1
	2011–2020	850	64.8
	2020-	62	4.7
	Not provided	4	0.3
During date	0–12 months	316	24.1
	13–24 months	295	22.5
	25–36 months	228	17.4
	37–48 months	180	13.7
	49–60 months	127	9.7
	60–months	115	8.8
	Not provided	51	3.9
Enrollment	0–99	657	50.1
	100–499	455	34.7
	500–999	100	7.6
	1,000-	94	7.2
	Not provided	6	0.5
Country	United States	678	51.7
	European Union	195	14.9
	Other	268	20.4
	Not provided	171	13
Interventions	Behavioral	787	60.0
	Drug	156	11.9
	Dietary Supplement	95	7.2
	Device& Procedure	75	5.7
	Other	199	15.2
Funded	NIH	222	16.9
	Industry	81	6.2
	U.S. Fed	33	2.5
	Other	976	74.4
Status	Completed	816	62.2
	Ongoing	306	23.3
	Stopped early	57	4.3
	Unknown status	133	10.1
Study results for primary completed trials (*n* = 959)	Has results	110	11.5
	Submit the results to the registry within 1 year of the primary completion date	5	0.5
Time from start to completion	≤1 year	316	24.1
	>1 year	945	72.0
	Not provided	51	3.9
Reason for termination	Poor enrollment	16	28.1
	Funding problems	8	14.0
	The Head of the study has left	6	10.5
	COVID-19	4	7.0
	Drug/device availability	2	3.5
	Study team's decision	2	3.5
	Other	8	14.0
	Not provided	11	19.3

Of the 1,312 identified childhood obesity trials, 57 trials (nearly 4.3%) were terminated early for various reasons, including 5 suspended, 34 terminated, and 18 withdrawn trials. The two most common reasons reported in the terminated trials were enrollment problems (*n* = 16, 28.1%) and funding problems (*n* = 8, 14.0%), followed by (Head of the study left) (*n* = 6, 10.5%), COVID-19 (*n* = 4, 7.0%) and Drug/device availability (*n* = 2, 3.5%). Of the total number of terminated trials, 11 trials (19.3%) did not report the reason for their early termination.

A total of 959 trials (73.1%) were completed before Jul. 29, 2020; nevertheless, only 110 trials (11.5%) submitted their results to the registry. Five trials (0.5%) submitted their results to the registry within 1 year from the primary completion date.

### Design Characteristics

The design characteristics of the selected trials are shown in Table 2. Of the 1,312 trials, less than half provided information regarding their phase, as most of them were nondrug trials. Randomized design (75.5%) and parallel assignment (72.1%) were most commonly utilized. Some form of masking was used in 528 trials (41.8%), of which 22.9% were blinded to the participants. The primary purpose of the childhood obesity trials was treatment (46.9%), followed by prevention (37.6%).

**Table 2:** Design characteristic of childhood obesity trials (*n* = 1,312).

**Items**	**Detail**	**Number**	**Percent**
Phases	Early phase 1	10	0.8
	Phase 1	31	2.4
	Phase 1|Phase 2	26	2.0
	Phase 2	59	4.5
	Phase 2|Phase 3	18	1.4
	Phase 3	56	4.3
	Phase 4	54	4.1
	Not applicable	1,058	80.6
Allocation	Randomized	991	75.5
	Non-randomized	148	11.3
	Not provided	173	13.2
Intervention model	Parallel assignment	946	72.1
	Single group assignment	220	16.8
	Crossover assignment	77	5.9
	Factorial assignment	48	3.7
	Sequential assignment	10	0.8
	Not provided	11	0.8
Masking	Single	305	23.2
	Double	93	7.1
	Triple	51	3.9
	Quadruple	99	7.5
	None (Open Label)	753	57.4
	Not provided	11	0.8
Primary purpose	Treatment	615	46.9
	Prevention	493	37.6
	Basic science	43	3.3
	Health services research	35	2.7
	Supportive care	32	2.4
	Diagnostic	21	1.6
	Screening	9	0.7
	Other	48	3.7
	Not provided	16	1.2

### Characteristics Associated With Early Trial Discontinuation

Univariable Cox regression analysis revealed a significant difference in the rate of early trial discontinuation between the different types of interventions, sample sizes, funding sources, and primary purposes (Table 3). Small trials (HR = 7.52, 95% CI: 3.53–16.04), drug trials (HR = 5.14, 95% CI: 2.96–8.91), industry trials (HR = 4.23, 95% CI: 2.12-8.44) and prevention trials (HR = 2.98, 95% CI: 1.43–6.21) were more frequently discontinued early than large trials, nondrug trials, NIH/other trials and treatment trials. In multivariable analysis, sample size and funding source remained significant determinants of trial discontinuation (Table 4). Trials primarily funded by industry were more likely to result in discontinuation compared with those funded by others (aHR = 3.09, 95% CI: 1.15–8.34, P = 0.026). Smaller trials were also more likely to be discontinued (aHR = 4.93, 95% CI: 2.02–12.01, *P* < 0.001). The subgroup analysis yielded consistent results (Table 5, Supplementary Table 1).

**Table 3:** Cox regression analysis of childhood obesity trials characteristics associated with early trial discontinuation.

**Variable**		**Univariable**		**Multivariable**	
		HR^*^(95%CI)	***P***	aHR^*^(95%CI)	***P***
Enrollment	0–100	7.52 (3.53–16.04)	**0.000**	4.93 (2.02–12.01)	**0.000**
	>100	Reference^*^		Reference	
Country	United States	1.71 (0.84–3.48)	0.137	1.61 (0.72–3.63)	0.249
	Non- united states	Reference		Reference	
Interventions	Non-drug	Reference		Reference	
	Drug	5.14 (2.96–8.91)	**0.000**	2.34 (0.92–5.92)	0.073
Funded	Industry	4.23 (2.12–8.44)	**0.000**	3.09 (1.15–8.34)	**0.026**
	Other	Reference		Reference	
Allocation	Randomized	1.43 (0.51–4.04)	0.499	1.37 (.39–4.89)	0.626
	Non-randomized	Reference		Reference	
Masking	None (Open Label)	0.97 (0.55–1.69)	0.906	1.24 (0.54–2.85)	0.604
	Yes	Reference		Reference	
Primary purpose	Treatment	Reference		Reference	
	Prevention	2.98 (1.43–6.21)	**0.004**	0.83 (0.34–2.00)	0.669
	Other	2.87 (1.14–7.23)	**0.026**	1.17 (0.37–3.70)	0.784

* *HR, hazard ratio; aHR, adjusted hazard ratio; P, P value. The bold values indicate P values which are statistically significant*.

*Reference: reference group, the category of the dummy variable excluded from the regression model*.

**Table 4:** Logistic regression analysis of childhood obesity trials characteristics associated with study results for completed trials.

**Variable**		**Univariable**		**Multivariable**	
		**OR (95%CI)**	***P***	**aOR (95%CI)**	***P***
Enrollment	0–100	1.31 (0.87–1.96)	0.196	1.11 (0.63–1.95)	0.725
	>100	Reference		Reference	
Country	United States	7.39 (3.78–14.47)	**0.000**	6.76 (3.32–13.78)	**0.000**
	Non- united states	Reference		Reference	
Interventions	Non-drug	Reference		Reference	
	Drug	5.14 (3.20–8.26)	**0.000**	6.62 (3.20–13.69)	**0.000**
Funded	Industry	2.19 (1.12–4.28)	**0.022**	0.59 (0.21–1.62)	0.303
	Other	Reference		Reference	
Allocation	Randomized	1.67 (0.78–3.54)	0.185	1.57 (0.55–4.44)	0.398
	Non-randomized	Reference		Reference	
Masking	None (Open Label)	0.80 (0.54–1.20)	0.281	0.73 (0.41–1.27)	0.262
	Yes	Reference		Reference	
Primary purpose	Treatment	Reference		Reference	
	Prevention	1.45 (0.93–2.25)	0.104	0.90 (0.49–1.65)	0.730
	Other	1.40 (0.76–2.60)	0.282	1.04 (0.40–2.70)	0.931

*OR, odds ratio; aOR, adjusted odds ratio; P, P value. The bold values indicate P values which are statistically significant*.

*Reference: reference group, the category of the dummy variable excluded from the regression model*.

**Table 5:** Risk factors for early discontinuation according to Cox regression model^*^.

**Variable**		**Univariable**		**Multivariable**	
		HR^*^(95%CI)	***P***	aHR^*^(95%CI)	***P***
Enrollment	0–100	10.44 (3.61–30.17)	**0.000**	6.22 (1.94–19.87)	**0.002**
	>100	Reference^*^		Reference	
Country	United States	1.60 (0.64–4.00)	0.315	1.35 (0.46–3.92)	0.586
	Non- united states	Reference		Reference	
Interventions	Non-drug	Reference		Reference	
	Drug	6.31 (2.92–13.64)	**0.000**	3.16 (0.78–12.89)	0.109
Funded	Industry	4.82 (1.96–11.87)	**0.001**	3.96 (1.08–14.53)	**0.038**
	Other	Reference		Reference	
Allocation	Randomized	1.66 (0.38–7.20)	0.495	2.37 (0.30–18.76)	0.414
	Non-randomized	Reference		Reference	
Masking	None (Open Label)	1.10 (0.52–2.34)	0.800	2.39 (0.74–7.74)	0.147
	Yes	Reference		Reference	
Primary purpose	Treatment	Reference		Reference	
	Prevention	3.21 (1.30–7.96)	**0.012**	1.10 (0.38–3.18)	0.862
	Other	1.52 (0.31-−7.54)	0.610	0.59 (0.07–5.27)	0.640

* *Subgroup analysis: group1: children only*.

*HR, hazard ratio; aHR, adjusted hazard ratio; P, P value. The bold values indicate P values which are statistically significant*.

*Reference: reference group, the category of the dummy variable excluded from the regression model*.

### Characteristics Associated With Reporting Results in the Registry

Univariable logistic regression showed significant differences in the rate of reporting results in the registry, according to different types of countries, interventions, and funding sources. United States trials (OR = 7.14, 95% CI: 3.04–16.7), drug trials (OR = 5.91, 95% CI: 2.61–13.36), and industry trials (OR = 7.70, 95% CI: 1.67–35.42) were more frequently reported than non-United States trials, nondrug trials, and NIH/other trials. Further multivariable logistic regression showed that United States trials were more likely to report results in ClinicalTrials.gov than all other trials (aOR = 6.76, 95% CI: 3.32–13.78). Drug trials were more likely to report results than Nondrug trials (aOR = 6.62, 95% CI: 3.20–13.69). The subgroup analysis yielded consistent results (Table 6, Supplementary Table 2).

**Table 6:** Risk factors for results according to Logistic regression model^*^.

**Variable**		**Univariable**		**Multivariable**	
		**OR (95%CI)**	***P***	**aOR (95%CI)**	***P***
Enrollment	0-100	1.44 (0.82–2.53)	0.203	1.18 (0.52–2.70)	0.695
	>100	Reference		Reference	
Country	United States	12.29 (3.76–40.19)	**0.000**	12.89 (3.57–46.51)	**0.000**
	Non- united states	Reference		Reference	
Interventions	Non-drug	Reference		Reference	
	Drug	8.65 (4.31–17.36)	**0.000**	14.79 (4.65–47.03)	**0.000**
Funded	Industry	3.50 (1.56–7.89)	**0.002**	1.28 (0.30–5.44)	0.740
	Other	Reference		Reference	
Allocation	Randomized	1.77 (0.62–5.10)	0.288	1.28 (0.31–5.29)	0.736
	Non-randomized	Reference		Reference	
Masking	None (Open Label)	0.77 (0.44–1.34)	0.350	0.78 (0.32–1.85)	0.566
	Yes	Reference		Reference	
Primary purpose	Treatment	Reference		Reference	
	Prevention	1.63 (0.88–3.04)	0.121	0.84 (0.35–2.01)	0.694
	Other	2.07 (0.91–4.71)	0.083	0.73 (0.13–4.12)	0.721

* *Subgroup analysis: group1: children only*.

*OR, odds ratio; aOR, adjusted odds ratio; P, P value. The bold values indicate P values which are statistically significant*.

*Reference: reference group, the category of the dummy variable excluded from the regression model*.

## Discussion

Our study showed that among clinicalTrials.gov registered childhood obesity-related intervention trials, trial unreported results were 88.5%, and 4.3% of trials were prematurely terminated. The factors that reduced the risk of unreported outcomes were US-registered clinical studies and drug intervention trials. Factors associated with reduced risk of early termination are NIH or other federal agency funding and large trials. The study also suggests that behavioral interventions play an important role in pediatric obesity clinical trials. Fewer than half of the trials provided information about their trial phase, and fewer than half used blinding.

Like obesity adults, obesity children have the right to receive the highest attainable standard of health care, but there is a lack of evidence regarding the safety and efficacy of treatments for overweight and obesity in children (9). For drug interventions, scientific data from only a limited number of short-term clinical trials are available, therefore, clinical trials with children are of the utmost importance (8–10). Ethical issues and policy requirements make it more difficult to recruit children, leading to the possible early termination of many clinical trials. Previous studies have found that pediatric clinical trials are terminated frequently (19, 20, 26). Thousands of children have participated in these trials, which means considerable inefficiency and a waste of financial resources (18). However, the early termination rate of pediatric obesity trials (4.3%) was lower than that of neonatal clinical trials, pediatric cardiovascular trials, pediatric psychological trials, etc. (21, 27, 28). This was an unexpected result, which may be due to a large number of obese children and the fact that most of the trial interventions are behavioral interventions that are easily accepted by patients, so clinical trials can better recruit patients. Furthermore, our analysis showed that industry-funded trials were more likely to stop early than government-funded or others, which may be because industry-funded clinical trials are more subject to financial and commercial influence than government-funded trials (21). Stefan et al. conducted a retrospective cohort study of pediatric randomized controlled trials in Switzerland, Germany, and Canada and found that the larger sample sizes (Enrollment>100) were less likely to stop early (29), which is consistent with our results. Perhaps because these trials were better organized from the start, the trial design systems built around experienced researchers may be better able to cope with recruitment challenges. In univariate analyses, drug trials were more likely to stop early, which may be due to the high ethical and regulatory requirements of drug trials for children, which may affect enrollment. Prevention trials are also risk factors for early termination, which is consistent with Turner et al. (30). The possible explanation is that prevention trials. One possible explanation is that prevention trials have a comparatively higher time cost to achieve clinical benefits than treatment trials (31). In addition, our analysis found that 11 of 57 (19.3%) intervention trials on childhood obesity did not report the reason for their early termination on ClinicalTrials.gov. The results reporting is especially important if the study is discontinued because of the detection of harm caused by the intervention. Trials involving children should always post reasons for early discontinuation on ClinicalTrials.gov (26). Studies have shown that failure to recruit sufficient numbers of participants and funding problems are the most common reasons for premature termination of clinical trials (32). Our study confirms these findings in childhood obesity trials.

The ethics and regulations of research involving children are widely discussed in the current literature (33–37); however, the reporting of results is ignored. Underreporting remains an important issue in the field of childhood obesity. Therefore, this issue should raise concerns about the overall quality of pediatric clinical trials. Despite the policy of the International Council of Medical Journal Editors (ICMJE), which recommends that all investigators report results (38), we found that the quality of results reported in the childhood obesity registry is still poor. These conclusions are consistent with Yang et al. (18). In clinical studies of mental health and neurology (30, 39). the reporting rates of clinical trial results were 42.3% and 32.2% respectively, both higher than our study results (11.5%). Food and Drugs Administration Amendments Act (FDAAA) and the Final Rule do not mandate that all studies report their results to the registry, which may account for the different reporting rates of study results (15). Similarly, USA-trials and drug trials were more likely to report results on ClinicalTrials.gov, which is related to the policy requiring reporting of the Food and Drugs Administration Amendments Act (FDAAA) and the Final Rule (15). In univariate analyses, industry-sponsored trials are more likely to report results, possibly because industry-sponsored trials more often have favorable results and conclusions (40).

### Recommendations for Future Studies

First, study protocols and designs should be sternly controlled by supervisors, with particular emphasis on feasibility analysis of the conditions and expected numbers of subjects to be enrolled in trials, to ensure that studies with both reliable sample estimation and feasibility are available to allow patients to be enrolled. Second, childhood obesity trials should be routinely monitored by policymakers for registration and results on ClinicalTrials.gov, while ensuring trials are discontinued only for legitimate reasons. Third, due to the particularity, stringent ethical requirements, and lack of commercial benefits of clinical trials in children, most results of adult trials cannot be directly extended to children (41). Therefore, researchers need to publish trial results in a timely manner, especially for pediatric diseases. Fourth, the issue of inadequate enrollment emphasizes the importance of establishing global networks to coordinate recruitment efforts. Therefore, when designing a study, if the patient population is geographically dispersed and limited, researchers should positively collaborate with both clinicians and patient groups in each region and communicate with potential participants to maximize the number of enrollments needed to complete the trial.

### Limitations

Our study has several limitations. First, this study only analyzed trials registered at ClincialTrials.gov, and our analysis did not include trials in other areas of pediatrics. Moreover, it should also be noted that information in the registry is provided by investigators and sponsors, and we were not able to check the degree of accuracy of the trial data. However, this issue is mitigated in part by automated data validity checks and manual review by ClinicalTrials.gov staff to ensure data reliability before public posting (42).

## Conclusions

In summary, the problem of unreported results in clinical trials of childhood obesity is serious, and the factors that reduced the risk of unreported outcomes were US-registered clinical studies and drug intervention trials. Furthermore, the factors associated with a reduced risk of early termination are NIH or other federal agency funding and large trials. Our findings may contribute to a better understanding of the etiologies of premature termination and unreported results, thus setting the stage for targeted approaches to improve clinical research.

## Data Availability Statement

The raw data supporting the conclusions of this article will be made available by the authors, without undue reservation.

## Author Contributions

LD and JH conceptualized and designed the study and reviewed and revised the manuscript. XW and YL collected and screened the data, carried out the initial analyses, drafted the initial manuscript, and reviewed and revised the manuscript. LY coordinated and supervised data collection and critically reviewed the manuscript for important intellectual content. All authors approved the final manuscript as submitted and agree to be accountable for all aspects of the work.

## Funding

This study was supported by the Key Program of Sichuan Provincial Science and Technology Department, China (No. 2019YFS0194) and the National Natural Science Foundation of China (No. 81873197, No. 72074161, and No.81403276).

## Conflict of Interest

The authors declare that the research was conducted in the absence of any commercial or financial relationships that could be construed as a potential conflict of interest.

## Publisher's Note

All claims expressed in this article are solely those of the authors and do not necessarily represent those of their affiliated organizations, or those of the publisher, the editors and the reviewers. Any product that may be evaluated in this article, or claim that may be made by its manufacturer, is not guaranteed or endorsed by the publisher.

## Abbreviations

NIH: national institutes of health
HR: hazard ratio
OR: odds ratio
CI: confidence interval
FDA: food and drug administration
NCT: national clinical trial
FDAAA: food and drug administration amendments act
RCT: randomized controlled trial
ICMJE: international council of medical journal editors.
