# Supplementary material for: Results Reporting and Early Termination of Childhood Obesity Trials Registered on ClinicalTrials.gov
Source: Front Pediatr. 2022 Mar 24;10:860610. doi: 10.3389/fped.2022.860610 (PMC8987712; doi:10.3389/fped.2022.860610)
Supplement: Supplementary file 1 [file Table_1.DOCX]

**Supplementary Table**

Supplementary Table 1 Risk factors for early discontinuation according to Cox regression model*

| Variable |  | Univariable | | Multivariable | |
| --- | --- | --- | --- | --- | --- |
|  |  | HR* (95%CI) | *P* | aHR* (95%CI) | *P* |
| Enrollment | 0-100 | 4.94(1.67-14.62) | **0.004** | 3.19(0.76-13.37) | 0.113 |
|  | ＞100 | Reference* |  | Reference |  |
| country | United States | 1.88(0.62-5.74) | 0.267 | 1.58(0.42-5.94) | 0.495 |
|  | Non- United States | Reference |  | Reference |  |
| Interventions | Non-drug | Reference |  | Reference |  |
|  | Drug | 4.67(2.06-10.59) | **0.000** | 2.78(0.68-11.42) | 0.156 |
| Funded | Industry | 3.57(1.21-10.50) | **0.021** | 1.79(0.36-8.95) | 0.477 |
|  | Other | Reference |  | Reference |  |
| Allocation | Randomized | 1.22(0.28-5.34) | 0.792 | 0.75(0.11-4.91) | 0.761 |
|  | Non-Randomized | Reference |  | Reference |  |
| Masking | None (Open Label) | 0.87(0.38-1.99) | 0.735 | 0.49(0.11-2.11) | 0.335 |
|  | Yes | Reference |  | Reference |  |
| Primary Purpose | Treatment | Reference |  | Reference |  |
|  | Prevention | 2.72(0.77-9.56) | **0.012** | 0.59(0.12-2.89) | 0.516 |
|  | Other | 3.92(1.00-15.29) | **0.049** | 2.09(0.39-11.18) | 0.390 |

* **Subgroup analysis:** group 2: both children and adults

HR: hazard ratio; aHR: adjusted hazard ratio

Reference: reference group, the category of the dummy variable excluded from the regression model.

Supplementary Table 2 Risk factors for results according to Logistic regression model*

| Variable |  | Univariable | | Multivariable | |
| --- | --- | --- | --- | --- | --- |
|  |  | OR* (95%CI) | *P* | aOR* (95%CI) | *P* |
| Enrollment | 0-100 | 1.11(0.62-2.00) | 0.727 | 1.11(0.49-2.49) | 0.808 |
|  | ＞100 | Reference* |  | Reference |  |
| country | United States | 5.34(2.32-12.27) | **0.000** | 4.38(1.79-10.68) | **0.001** |
|  | Non- United States | Reference |  | Reference |  |
| Interventions | Non-drug | Reference |  | Reference |  |
|  | Drug | 3.05(1.58- 5.88) | **0.001** | 3.50(1.33-9.26) | **0.011** |
| Funded | Industry | 1.01(0.29-3.56) | 0.984 | 0.25(0.05-1.39) | 0.113 |
|  | Other | Reference |  | Reference |  |
| Allocation | Randomized | 1.53(0.52-4.52) | 0.443 | 2.30(0.46-11.47) | 0.310 |
|  | Non-Randomized | Reference |  | Reference |  |
| Masking | None (Open Label) | 0.89(0.50-1.59) | 0.703 | 0.81(0.38-1.75) | 0.596 |
|  | Yes | Reference |  | Reference |  |
| Primary Purpose | Treatment | Reference |  | Reference |  |
|  | Prevention | 1.12(0.59-2.13) | 0.732 | 0.92(0.38-2.23) | 0.856 |
|  | Other | 0.79(0.31-2.03) | 0.629 | 1.12(0.34-3.71) | 0.852 |

* **Subgroup analysis:** group 2: includes both children and adults

OR: odds ratio; aOR: adjusted odds ratio

Reference: reference group, the category of the dummy variable excluded from the regression model.
